# Predicting Emergency Department “Bouncebacks”: A Retrospective Cohort Analysis

**DOI:** 10.5811/westjem.2019.8.43221

**Published:** 2019-10-16

**Authors:** Juan Carlos C. Montoy, Joshua Tamayo-Sarver, Gregg A. Miller, Amy E. Baer, Christopher R. Peabody

**Affiliations:** * University of California, San Francisco, Department of Emergency Medicine, San Francisco, California; † Vituity Healthcare, Emeryville, California

## Abstract

**Introduction:**

The short-term return visit rate among patients discharged from emergency departments (ED) is a quality metric and target for interventions. The ability to accurately identify which patients are more likely to revisit the ED could allow EDs and health systems to develop more focused interventions, but efforts to reduce revisits have not yet found success. Whether patients with a high number of ED visits are at increased risk of a return visit remains underexplored.

**Methods:**

This was a population-based, retrospective, cohort study using administrative data from a large physician partnership. We included patients discharged from EDs from 80 hospitals in seven states from July 2014 – June 2016. We performed multivariable logistic regression of short-term return visits on patient, visit, hospital, and community characteristics. The primary outcome was the proportion of patients who had a return visit within 14 days of an index ED visit.

**Results:**

Among 6,699,717 index visits, the overall risk of 14-day revisit was 12.6%. Frequent visitors accounted for 18.7% of all visits and 40.2% of all 14-day revisits. Frequent visitor status was associated with the highest odds of a revisit (odds ratio [OR] 3.06; 95% confidence interval [CI], 3.041 – 3.073). Other predictors of revisits were cellulitis (OR 2.131; 95% CI, 2.106 – 2.156), alcohol-related disorders (OR 1.579; 95%CI, 1.548 – 1.610), congestive heart failure (OR 1.175; 95% CI, 1.126 – 1.226), and public insurance (Medicaid OR 1.514; 95% CI, 1.501 – 1.528; Medicare OR 1.601; 95% CI, 1.583 – 1.620).

**Conclusion:**

Previous ED use – even a single previous visit – was a stronger predictor of a return visit than any other patient, hospital, or community characteristic. Clinicians should consider previous ED use when considering treatment decisions and risk of return visit, as should stakeholders targeting patients at risk of a return visit.

## INTRODUCTION

Short-term outcomes – including return emergency department (ED) visits – after discharge from the ED are used as internal quality metrics, as short-term revisits might represent medical errors or failures in care.^1^–^3^ Although interventions to reduce return visits have largely been unsuccessful,^4^ it is possible that these efforts did not adequately target high-risk patients. Related literature is focused on patients who have a pattern of repeat ED use; however, surprisingly, the degree to which these frequent users contribute to short-term revisits remains unknown.

The ability to accurately identify which patients are more likely to revisit the ED could improve treatment and disposition decisions, and also allow EDs and health systems to develop more focused interventions. Previous work has identified some predictors of return visits,^5^–^7^ although these studies are limited by investigating only a subset of patients,^8^–^11^ restriction to one or few sites,^12^–^15^ focus on non-U.S. hospitals,^16^–^18^ reliance on complicated instruments,^19^–^22^ focus on medical errors,^23^ focus on admissions,^24^,^25^ or use of overly-broad definition of discharge failure.^26^

We used a unique dataset with encounter-level data to evaluate the predictors of return visits. Our goal was to identify which patient demographics and medical conditions were most associated with short-term revisits. In addition, we hypothesized that frequency of recent previous visits – specifically, number of visits within the previous six months – would have a stronger association with return visits than other patient characteristics (including initial diagnosis), and that this pattern would be observed even after controlling for hospital and community characteristics.

## METHODS

### Design

We conducted a retrospective study of patients visiting 80 hospitals in seven states from July 1, 2014 – June 30, 2016. In addition, we included data for six months prior and 30 days after the start and end dates, respectively, in order to observe activity around the index visit, giving a total range from January 1, 2014 – July 30, 2016. Encounter data were obtained from Vituity, a multistate physician partnership that contracts with hospitals to provide ED provider staffing. During the study period, Vituity (then known as California Emergency Physicians [CEP] America) provided staffing for 121 EDs in 13 states. Only sites with full contracts and data availability for the entire study period were included. The study received institutional review board approval.

### Study setting and population

All patient encounters were eligible for inclusion. We excluded encounters as potential index visits if patients eloped (left prior to discharge from the ED), died while in the ED, or were transferred to another facility.

### Methods and measurements

Data were recorded in the medical record at each hospital. Vituity collects this data through monthly electronic data feeds by its medical billing company, MedAmerica Billing Systems, Inc, which stores records in Application System / 400 and PostgreSQL. Patient visits were linked through Medical Person Identification number – a unique patient identifier derived by an algorithm taking into consideration patient name, date of birth, Social Security number, and address. This methodology allowed for linkage across sites, although visits at non-Vituity sites were not observable. Any visit had the potential to be defined as an index visit.

Patient characteristics included age, sex, insurance type (Medicare, Medicaid, commercial, or other), and the number of ED visits they had in the six months prior to the index visit. We reduced previous ED visits to an indicator variable for two or more previous visits in order to identify a characteristic that was easily observed and easy to apply to patients in real time.

**Population Health Research Capsule**

What do we already know about this issue?

*Short-term revisits to emergency departments (ED) and frequent ED utilization have each been studied, but the relationship between the two remains underexplored*.

What was the research question?

*To identify which patient characteristics, including recent frequent use, were most associated with short-term revisits*.

What was the major finding of the study?

*Recent frequent use was a stronger predictor of a revisit than other patient, hospital, and community characteristics*.

How does this improve population health?

*Clinicians should consider previous ED use when considering treatment decisions and risk of return visit, as should stakeholders targeting these high-risk patients*.

Visit characteristics included acuity level, primary diagnosis, and Charlson comorbidity index. Primary diagnoses were categorized using *International Classification of Disea*ses, 9^th^ and 10^th^ revisions (ICD-9 and 10 codes according to Agency for Health Care Research and Quality (AHRQ) Clinical Categorization Software (CCS) categories. These categories were developed and defined by the Healthcare Cost and Utilization Project (HCUP), under the AHRQ, and this scheme has been used in a number of studies.^27^,^28^ Because of the large number of categories, we further restricted diagnoses to the diagnoses that had at least 10,000 observations and were associated with 14-day revisits in bivariate analysis; among these, we included the five most common diagnoses for index visits and for revisits. Charlson comorbidity index was calculated for all visits based on up to 12 separate ICD codes per visit (Appendix A and B; Tables S1, S2).^29^,^30^

Hospital characteristics included size (volume for 2015), and turnaround time to discharge (TAT-D) for 2015. TAT-D is a quality metric measuring the median time between patient arrival and discharge at the hospital level for a given year. Volume was broken into four categories as defined by the Centers for Medicare & Medicaid Services: fewer than 20,000 encounters = low volume; 20,000 – 39,999 encounters = medium volume; 40,000 – 59,999 encounters = high volume; and greater than or equal to 60,000 encounters = very high volume.

Community characteristics were comprised of zip code and county-level characteristics: median household income for zip code, number of hospitals per 1000 population in the county, and county. Zip code median household income was broken into quartiles based on the following: less than or equal to $44,168 = low income; $44,169 – $53,647 = medium income; $53,648 – $66,275 = high income; and greater than or equal to $66,276 = very high income.^31^

Physician characteristics included provider type: doctor (MD or DO) or advanced practice provider (APP; ie, physician assistant or nurse practitioner). We excluded from the study providers working for the firm for fewer than 60 days within the study period or accounting for fewer than 60 encounters. To test whether there was a different likelihood in return visit according to acuity level, we included interaction terms between MD/DO and acuity level; given the difference in scope of practice for APP, interactions between APP and acuity level were not modeled.

### Outcomes

The primary outcome was the proportion of patients who had a return visit within 14 days of an index ED visit. Secondary outcomes included proportion of patients with a revisit within 3, 7, and 30 days of discharge; and likelihood of revisit according to number of visits in the six months prior to the index visit. We selected these time horizons due to use of each of these in the literature and their policy implications.^32^

### Analysis

The primary outcome was the risk of return visit within 14 days. We calculated the proportion of patients who returned to the ED within 3, 7, 14, and 30 days after the index visit. We performed a multivariable logistic regression, regressing return visit on patient, physician, hospital, and community characteristics. Hospitals and counties were estimated to have random effects. Standard errors were clustered at the physician, hospital, and county levels. In sensitivity analyses, we estimated the model each of three ways: i) for a subset of the data that excluded patients aged <18 years; ii) using different thresholds for frequent visitor (one or more and three or more visits in the previous six months); and iii) using different time horizons for repeat visit (3, 7, and 30 days); we also conducted analyses for all combinations of frequent visitor threshold and time horizon. Analyses were conducted using SAS software, Version 9.4 (SAS Institute, Cary, NC) of the SAS System for Windows.

## RESULTS

**Figure 1:**
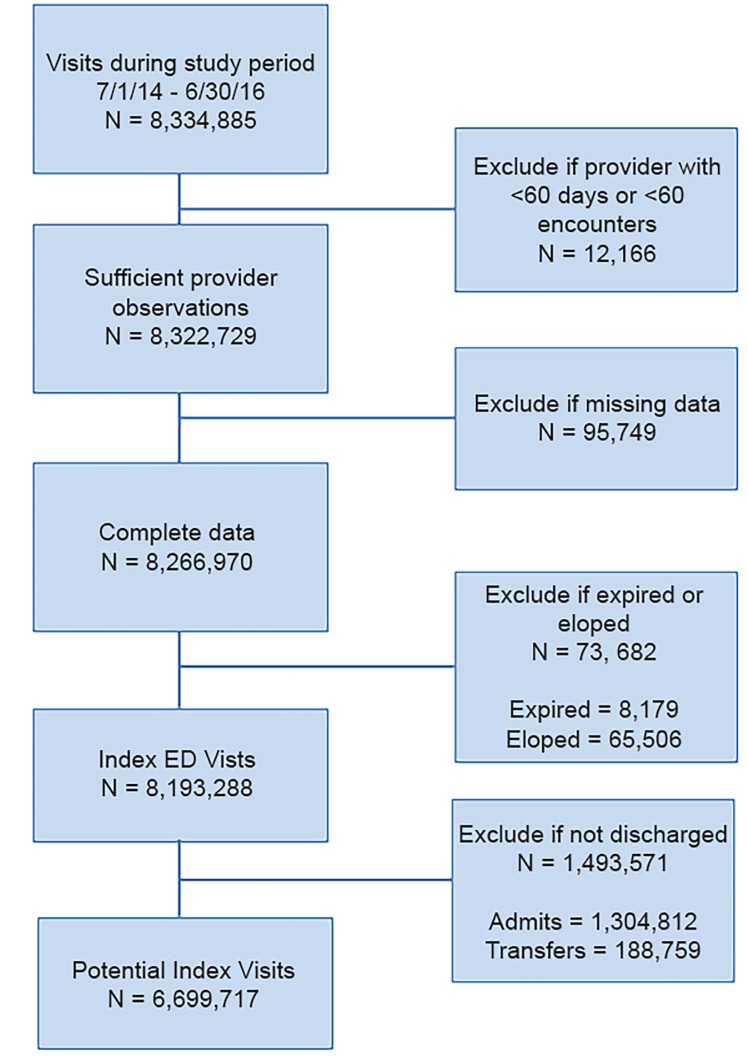
CONSORT-like flow diagram. *ED*, emergency department.

Over the study period, there were 8,334,885 index encounters. After excluding visits resulting in a disposition other than discharge and excluding visits with missing data, the total sample size was 6,699,717 (Figure 1). Table 1 shows the patient, visit, hospital, and physician characteristics at index visit for all encounters, and stratified by discharge vs admission. These descriptive statistics are also shown for encounters resulting in a 14-day return and for those who returned and were admitted to the hospital.

In the multivariate model including patient, hospital, and community characteristics (Table 2), the highest predictor of return visit within 14 days was whether or not the patient had two or more visits in the previous six months: OR = 3.06 (95% confidence interval [CI], 3.041 – 3.073). Men and patients with Medicare or Medicaid insurance were more likely to have 14-day revisits, as were patients with a primary diagnosis of alcohol-related disorder; complication of device, implant or graft; congestive heart failure; and schizophrenia and other psychotic disorders.

As a sensitivity analysis, we estimated the same model among adult patients only and found the results did not show any meaningful differences. Further, we repeated the analysis for each definition of frequent visitor definition (one or more and three or more previous visits) and time horizons (3-, 7-, and 30-day revisits), and each combination of frequent visitor and time horizon. Skin and subcutaneous tissue infections (SSTI) were the strongest predictor of three-day revisits for each of the definitions of frequent visitor, followed by frequent visitor as the next largest association. In all other specifications, frequent visitor was the factor with the strongest association with revisits.

**Figure 2:**
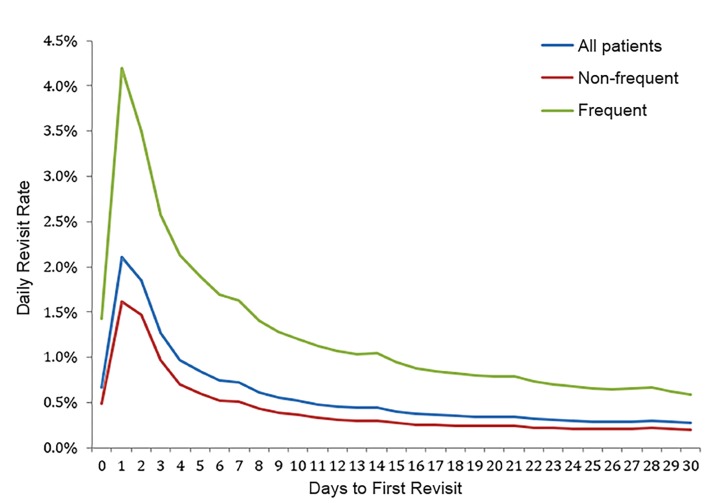
Percentage of patients with an emergency department revisit. Percentage of patients revisiting the emergency department according to day after the index visit for all patients, and separately for each of frequent and non-frequent visitors.

There were 476,665 frequent visitors, who had a total of 1,251,082 visits, of which 340,381 were 14-day revisits. While frequent visitors represent 10.7% of all patients, they accounted for 18.7% of all encounters and 40.2% of all 14-day revisits. They were more likely to have a return visit at all times as compared to non-frequent visitors. Figure 2 demonstrates the percentage of patients revisiting the ED according to day after the index visit.

The blue line represents all patients and shows that revisits peak on days one and two, and steadily decline thereafter, with slight peaks at days 7 and 14. The red line shows the revisit rate for patients with no or one visit in the six months prior to the index visit; as with all patients, the revisit rate peaks on days 1–2 and declines thereafter, dropping to below 0.3% by day 14.

Patients defined as frequent visitors have revisits peaking on day 1 and decrease thereafter. The daily revisit rate for frequent visitors declines to a value of about 1.0% at 14 days, after which the revisit percentage decreases by less than 0.1% for each subsequent day. Encounters showing 0 days to first revisit reflect patients who returned to the ED on the same day as their index visit. Same day revisits represented 3.7% of the total encounters with an associated revisit. Frequent visitors had a significantly higher risk of a 14-day return visit resulting in admission than non-frequent visitors (OR 2.89; 95% CI, 2.86 – 2.93).

Table 3 shows the unadjusted proportion of encounters resulting in return at 3 and 14 days according to different thresholds defining frequent visitor. For each threshold number of visits in the preceding six months, the unadjusted risk of return visit was more than double among frequent visitors as compared to non-frequent visitors. The remainder of the analysis uses two or more previous visits as the threshold defining frequent visitor, unless otherwise specified.

## DISCUSSION

This retrospective analysis of almost seven million patient visits found that recent previous ED visits was the strongest predictor of an ED return visit. This finding held true across multiple cutoffs defining frequent use, and also under both univariate analysis and a multivariate model including patient, visit, hospital, and county characteristics. Along with recent frequent use, public insurance and three diagnoses (cellulitis, alcohol-related disorders, and congestive heart failure) were associated with an increased risk of a return visit. This suggests that our understanding of short-term revisits could be informed by considering frequency of ED use.

A parallel thread in the literature has investigated frequent users and interventions designed to decrease ED use.^25^,^33^,^34^ Previous studies have evaluated predictors of ED revisit using patient-level data such as age, sex, race, insurance status, and diagnosis at initial ED visit, as well as hospital-level data. Surprisingly, the relationship between frequent ED use and risk of revisit after discharge is poorly characterized.^35^ Further, there is no consensus on what defines “frequent,” with definitions ranging from 2–12 visits per year.^36^–^41^ We had the striking finding that even one previous visit increased risk of return by a clinically-significant margin. This finding held true even when accounting for patient, visit, hospital, and community characteristics. Our definition focused on visits within the previous six months because other work has shown that episodes of frequent ED use are usually self-limited,^42^ which suggests that the recent past is more relevant to current health and risk of short-term return visit.

A second, related finding is that the threshold used to define frequent visitors is arbitrary with respect to risk of return visit. In the hope of informing the wide range in the literature on the number of visits or length of time used to define frequent users,^31^,^33^ we considered our definition of frequent user in relation to risk of return visit. We had the surp finding that *any* number of previous visits used to define frequent vs non-frequent ED users predicted an increased risk of revisit. Given that the reason to label certain patients as frequent visitors is often in order to identify them for interventions, future work may consider an outcome-based definition of frequent users and define the term “frequent” with a qualifier – eg, with respect to propensity to revisit after a visit, risk of becoming a persistent frequent user, or risk of death.

As with existing literature, we transformed the number of previous visits from a continuous variable to a binary one. This has the disadvantage of losing some information, but is standard in the literature regarding frequent ED use, and can easily be applied in the midst of clinical practice.^31^–^39^ Our sensitivity analysis demonstrated that any threshold was significantly associated with return visits, suggesting that knowing whether a patient had four vs three previous visits would provide marginally more information than simply knowing the patient had more than two previous ED visits.

As with the definition of frequent user, the time to return visit defining a return visit is somewhat arbitrary. While the risk of return visit is highest on the first day following the ED visit, the risk gradually decreases and, as found previously by Rising et al., there is no clear timeline that defines a return visit.^43^ This finding may suggest something other than inadequate care at the index visit is the driving factor for most short-term revisits, and that both frequent use and revisits may simply be proxies for certain patients with increased healthcare-seeking behavior. Further complicating this issue is that patients may be instructed to return to the ED for a re-evaluation. Thus, an ED in a setting with limited outpatient resources might appear to give poor care as measured by revisits when in fact it serves to provide follow-up care that patients otherwise would not obtain.

Despite the variation in the literature and thus our broad range of models, we consistently found that the strongest predictor of a revisit is a high number of previous visits. This finding held true in our sensitivity analysis using different thresholds for number of previous visits and also days after index visit. The observation that previous visits predicts future visits may seem obvious or mechanical, but it does not necessarily follow that a patient with one or two visits in the prior six months would be at double the risk of a revisit within three days. Further, that this relationship was stronger than any other patient, hospital, or community characteristic is an important finding that has been overlooked in the literature regarding revisits. In fact, it appears that the literature on frequent visitors and the literature regarding revisits have to this point largely functioned in parallel and have not yet begun to inform each other.

Whether frequent users are merely frequently-ill people, and whether sicker patients are at increased risk of short-term revisits deserves future research. Likewise, future work should investigate the extent to which patients are frequent users because they received poor care or face limitations in their ability to obtain outpatient resources, the extent to which revisits are avoidable, and the degree to which frequent use persists over time. Understanding the extent to which follow-up with primary care, referrals to specialists, and ability to obtain further evaluation such as advanced imaging, cardiac stress test, or even a wound check is essential to understanding why patients return to the ED.

## LIMITATIONS

The data for this study were obtained from a single multistate physician partnership and do not necessarily generalize to other providers or provider groups, or to other populations. However, the sample size was large and spans many cities and rural areas across several states, includes a broad set of hospital owner types, a large range of hospital sizes, and both teaching and non-teaching hospitals. This source of data may lead to a biased sample with respect to patient population, hospital characteristics, and provider characteristics. In particular, the income distribution is narrower than the distribution for the entire U.S., so the patient population could have a lower proportion of low- and high-income patients than typical for the U.S. We addressed these potential sources of bias by controlling for patient demographics, patient insurance, and local income; hospital characteristics including volume and a performance metric, and clinician degree.

Second, because not all hospitals within a region were observed, measures of frequent visitors and repeat visits may underestimate the actual numbers of frequent visitors and repeat visits, as patients may have gone to another ED either prior to or after the observed index visit. This limitation is typical of this research,^12^–^15^ and in this dataset patients were linked across hospitals, although this was limited to the hospitals served by this company. Thus, it is unknown whether patients had an unobserved revisit at another ED, or whether what was considered an index visit actually represented a revisit after an initial visit at another ED. Next, we were unable to distinguish between planned and unplanned return visits. Thus, a patient who is instructed to return for a check over the weekend to ensure their illness is improving, for example, would appear to be a revisit, but this should not imply that their initial treatment was inadequate or inappropriate in any way. Research using administrative datasets, such as HCUP, likewise suffers from this limitation.

Finally, as with related research, this study does not identify the extent to which high rates of frequent visits and revisits are driven by patient factors, ED care, or non-ED healthcare resources. This analysis was limited in its ability to examine patient psychosocial attributes or local resources, which are likely to contribute to ED visits and revisits, although we did consider proxies for access to care: patient insurance and community-level factors such as income and number of hospitals in the county.

## CONCLUSION

In our study of 6.7 million patients across seven states from 2014 to 2016 we found that a high number of ED visits – as defined by *any* threshold – within the previous six months are not only a significant predictor of short-term ED revisits, but have a stronger association than any other observable variable assessed in this study. The number of recent visits is an easily-obtained value that can be used in real-time by physicians, social workers, and case managers, and the threshold number of recent visits can be chosen by any ED to optimize how it deploys resources to prevent short-term revisits. In addition, the result here suggests a relationship between two parallel threads of literature – that regarding frequent users and short-term revisits – that has thus far gone largely unnoticed and deserves further attention.

## Supplementary Information



## Figures and Tables

**Table 1:** Patient, visit, hospital, and community characteristics.

Characteristic	Index ED visit	Index discharge	Index admitted	Return visit	Return and admitted
Number of patients	8,193,288	6,699,717	1,493,571	846,759	135,735
Patient factors					
Age (median, IQR)	39 (22–59)	34 (20–53)	62 (46–77)	40 (25–58)	56 (38–73)
Sex (female)	55.4%	56.3%	51.7%	55.3%	54.3%
Insurance					
Commercial	19.2%	19.8%	16.5%	12.2%	13.9%
Medicaid	47.0%	51.6%	26.1%	56.1%	36.2%
Medicare	23.8%	17.5%	51.9%	24.3%	45.5%
Other	10.0%	11.1%	5.5%	7.4%	4.3%
Frequent visitor	19.5%	18.7%	23.1%	40.2%	40.4%
Visit Factors					
E&M level					
1	0.4%	0.5%	0.0%	0.5%	0.2%
2	0.9%	1.1%	0.0%	1.2%	0.6%
3	36.8%	44.9%	0.2%	38.8%	18.8%
4	24.5%	29.6%	1.8%	31.9%	32.8%
5	34.6%	23.7%	83.6%	27.3%	46.9%
Critical care	2.8%	0.3%	14.4%	0.3%	0.7%
Primary Diagnosis					
Abdominal pain	7.4%	8.1%	4.3%	9.5%	10.8%
Alcohol-related	0.9%	1.0%	0.6%	1.7%	1.6%
Device or graft malfunction	0.3%	0.2%	0.3%	0.5%	0.8%
Congestive heart failure	0.8%	0.2%	3.3%	0.4%	1.2%
Schizophrenia	0.5%	0.3%	1.7%	0.5%	0.8%
SSTI	2.7%	2.8%	2.4%	4.9%	3.7%
Charlson comorbidity index	12.0%	7.0%	32.0%	10.0%	19.0%
Advanced practice provider	37.8%	44.1%	9.7%	39.7%	23.0%
Hospital					
ED volume (year)					
<20,000	2.3%	2.4%	1.9%	2.5%	2.2%
20,000–39,999	16.9%	17.3%	15.3%	17.5%	15.8%
40,000–59,999	19.3%	19.4%	19.1%	20.0%	20.3%
≥60,000	61.4%	60.9%	63.7%	60.1%	61.7%
Time until discharge (low)	82.8%	83.6%	79.3%	84.8%	81.9%
Community characteristics					
Median income for zip code, quartiles					
<$44,169	24.5%	25.1%	22.1%	26.9%	23.9%
$44,169 – $53,647	24.6%	25.0%	23.1%	25.4%	24.0%
$53,648 – $66,275	24.9%	25.0%	24.8%	25.2%	25.2%
>$66,275	25.9%	25.0%	30.0%	22.5%	27.0%
Hospitals per 1,000 persons (county)	150.3	149.6	153.6	147.1	150.1

*IQR*, interquartile range; *ED*, emergency department; *E&M level*, Evaluation and Management CPT codes; 1 is lowest acuity, critical care is highest acuity; *SSTI*, skin and subcutaneous tissue infection.

Frequent visitor is defined as two or more visits in the previous six months. Time until discharge is an indicator for median time until discharge less than or equal to 200 minutes.

**Table 2:** Multivariable regression results: 14-day revisits.

Effect	Odds ratio (95% confidence interval)
Patient characteristics	
Age	1.035 (1.034 – 1.036)
Age^2^	1.000 (0.999 – 1.000)
Age^3^	1.000 (1.000 – 1.000)
Male	1.12 (1.115 – 1.126)
Insurance Type (ref=other)	
Commercial	0.94 (0.930 – 0.940)
Medicaid	1.514 (1.501 – 1.528)
Medicare	1.601 (1.583 – 1.62)
Frequent visitor	3.057 (3.041 – 3.073)
Visit characteristics	
Primary Diagnosis (ref=other diagnosis)	
Abdominal pain	1.162 (1.152 – 1.172)
Alcohol-related disorders	1.579 (1.548 – 1.61)
Congestive heart failure	1.175 (1.126 – 1.226)
Complication of device, implant, or graft	1.576 (1.519 – 1.634)
Schizophrenia and other psychotic disorders	1.62 (1.563 – 1.68)
Skin and subcutaneous tissue infections	2.131 (2.106 – 2.156
Evaluation & Management Level (ref=1)	
2	1.194 (1.136 – 1.253)
3	1.028 (0.987 – 1.071)
4	1.152 (1.106 – 1.201)
5	1.241 (1.190 – 1.295)
CC	1.145 (0.893 – 1.467)
Charlson comorbidity index	1.194 (1.092 – 1.108)
Hospital characteristics	
ED volume (ref=low)	
Medium	1.027 (1.190 – 1.295)
High	1.037 (0.893 – 1.467)
Very High	1.035 (0.983 – 1.142)
Time to discharge (ref=low)	0.939 (0.874 – 1.009)
Provider characteristics	
MD or DO provider type (ref = APP)	1.187 (1.108 – 1.272)
Community characteristics	
Number of hospitals in county per 1,000 people	0.999 (0.998 – 1.000)
Income category (ref=low)	
Medium	0.994 (0.987 – 1.002)
High	1.001 (0.993 – 1.008)
Very High	0.947 (0.939 – 0.956)

*CI*, confidence interval; Ref, reference; *ED*, emergency department; *MD*, medical doctor; *DO*, doctor of osteopathic medicine; *NP*, nurse practitioner; *PA*, physician assistant; *CC*, critical care; *APP*, advanced practice provider (NP or PA).

**Table 3:** Risk of return according to previous visits.

Frequent visitor threshold (# of visits in previous 6 months)	3-day revisit		14-day revisit	
	Non-frequent	Frequent	Non-frequent	Frequent
1 or more*	4.10%	8.81%	8.11%	20.09%
2 or more*	4.54%	11.70%	9.29%	27.21%
3 or more*	4.82%	14.69%	10.05%	34.18%
4 or more*	5.01%	17.66%	10.57%	40.74%

* The result from each z-test testing the proportion of non-frequent versus frequent patients with a 3-day or 14-day revisit was statistically significant at the p < 0.001 level for each of the eight pair-wise comparisons.
