# Supplementary material for: Predicting Emergency Department “Bouncebacks”: A Retrospective Cohort Analysis
Source: West J Emerg Med. 2019 Oct 16;20(6):865–74. doi: 10.5811/westjem.2019.8.43221 (PMC6860392; doi:10.5811/westjem.2019.8.43221)
Supplement: Supplementary file 1 [file wjem-20-865-s001.docx]

**Predicting Emergency Department “Return Visits”:**

**A Retrospective Cohort Analysis**

**Appendix**

Table of Contents

1. Primary diagnoses classification
2. Most frequent clinical classifications: index visit and revisit
3. Dataset: Missing data

**Appendix A: Primary Diagnoses Output for 14-Day Revisits**

Primary Diagnosis, or CCS Category, had 272 levels--far too many to include in the full model. Therefore, we pre-specified that we would include, as indicator variables, the five levels with the highest odds of 14-day revisit which had both volume greater than 10,000 encounters (top third of all categories) and significant (p-value > 0.05) predictive power. We decided our reference for the univariate model would be the category with the highest volume for both Index and Revisits (Abdominal Pain) and that we would also include this level as one of our predictor variables.

After subsetting on these criteria, 20 primary diagnoses remained. Our top categories were: complication of device, implant, or graft; schizophrenia and other psychotic disorders; congestive heart failure; skin and subcutaneous tissue infections; and alcohol-related disorders.

**Appendix B: Most frequent clinical classifications**

**Appendix Table S1: Index Visit Most Frequent Clinical Classifications**

Index Visit (n=6,699,717)

| **CCS Category** | **Count** | **Percent** |
| --- | --- | --- |
| Abdominal pain | 542,827 | 8.10% |
| Other upper respiratory infections | 362,149 | 5.41% |
| Sprains and strains | 285,535 | 4.26% |
| Nonspecific chest pain | 253,300 | 3.78% |
| Superficial injury; contusion | 249,120 | 3.72% |
| Spondylosis; intervertebral disc disorders; other back problems | 228,593 | 3.41% |
| Other injuries and conditions due to external causes | 206,094 | 3.08% |
| Skin and subcutaneous tissue infections | 184,550 | 2.75% |
| Headache; including migraine | 183,518 | 2.74% |
| Urinary tract infections | 183,215 | 2.73% |

**Appendix Table S2: Return Visit Most Frequent Clinical Classifications**

Return Visit (n=846,759)

| **CCS Category** | **Count** | **Percent** |
| --- | --- | --- |
| Abdominal pain | 80,818 | 9.54% |
| Skin and subcutaneous tissue infections | 41,647 | 4.92% |
| Spondylosis; intervertebral disc disorders; other back problems | 32,518 | 3.84% |
| Other upper respiratory infections | 31,729 | 3.75% |
| Nonspecific chest pain | 29,028 | 3.43% |
| Headache; including migraine | 26,427 | 3.12% |
| Urinary tract infections | 24,236 | 2.86% |
| Superficial injury; contusion | 20,914 | 2.47% |
| Sprains and strains | 20,671 | 2.44% |
| Open wounds of extremities | 19,629 | 2.32% |

**Appendix C**

**Appendix Table S3: Variables Containing Missing Data**

|  | Number of Missing Encounters | Percent of Total Encounters |
| --- | --- | --- |
| **Patient factors** |  |  |
| Age* | 525 | 0.01% |
| Median Income by zip code** | 287 | 0.00% |
| **Medical pathology** |  |  |
| E&M level | 54,608 | 0.66% |
| Primary diagnosis | 1,977 | 0.02% |
| Charlson comorbidity | 537 | 0.01% |
| **Provider factors** |  |  |
| Provider*** | 264 | 0.00% |
| **TOTAL** | **58,198** | **0.70%** |

*Age over 110 years old was considered “bad data”/missing.

**Number of initial missing values for median household income by zip code was 2,719. After imputing with median income by state, there were 287 missing values corresponding to a median income by zip code of $10,000 or lower, which was considered “bad data”/missing.

*** Providers working for the firm for fewer than 60 days within the study period or accounting for fewer than 60 encounters were excluded from the study
